# Bis(diisopropyl­ammonium) tetra­chloridocuprate(II)

**DOI:** 10.1107/S1600536812004928

**Published:** 2012-02-10

**Authors:** Jie Xu

**Affiliations:** a College of Chemistry and Chemical Engineering, Southeast University, Nanjing 210096, People’s Republic of China

## Abstract

In the title mol­ecular salt, (C_6_H_16_N)_2_[CuCl_4_], the Cu^II^ ion adopts an extremely distorted tetra­hedral coordination geometry. All the ammonium H atoms are involved in N—H⋯Cl hydrogen bonds, which serve to link the cations and anions into chains propagating along the *c*-axis direction.

## Related literature

For background to mol­ecular ferroelectric crystals, see: Fu *et al.* (2011 ▶).
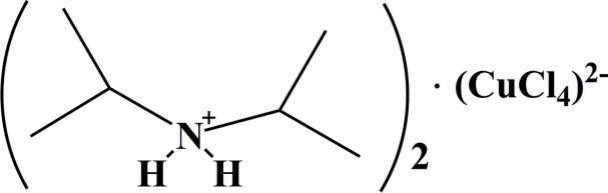


## Experimental

### 

#### Crystal data

(C_6_H_16_N)_2_[CuCl_4_]
*M* *_r_* = 409.74
Monoclinic, 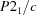

*a* = 10.541 (2) Å
*b* = 14.402 (3) Å
*c* = 14.641 (6) Å
β = 114.38 (2)°
*V* = 2024.5 (10) Å^3^
*Z* = 4
Mo *K*α radiation
μ = 1.60 mm^−1^
*T* = 298 K
0.10 × 0.03 × 0.03 mm

#### Data collection

Rigaku Mercury2 CCD diffractometer
Absorption correction: multi-scan (*CrystalClear*; Rigaku, 2005 ▶) *T*_min_ = 0.910, *T*_max_ = 1.000
20644 measured reflections
4637 independent reflections
3904 reflections with *I* > 2σ(*I*)
*R*_int_ = 0.036

#### Refinement

*R*[*F* ^2^ > 2σ(*F* ^2^)] = 0.035
*wR*(*F* ^2^) = 0.081
*S* = 1.10
4637 reflections
180 parameters
H-atom parameters constrained
Δρ_max_ = 0.50 e Å^−3^
Δρ_min_ = −0.41 e Å^−3^

### 

Data collection: *CrystalClear* (Rigaku, 2005 ▶); cell refinement: *CrystalClear*; data reduction: *CrystalClear*; program(s) used to solve structure: *SHELXS97* (Sheldrick, 2008 ▶); program(s) used to refine structure: *SHELXL97* (Sheldrick, 2008 ▶); molecular graphics: *SHELXTL* (Sheldrick, 2008 ▶); software used to prepare material for publication: *SHELXTL*.

## Supplementary Material

Crystal structure: contains datablock(s) I, global. DOI: 10.1107/S1600536812004928/hb6624sup1.cif

Structure factors: contains datablock(s) I. DOI: 10.1107/S1600536812004928/hb6624Isup2.hkl

Additional supplementary materials:  crystallographic information; 3D view; checkCIF report

## Figures and Tables

**Table:** 

Cu1—Cl1	2.2419 (9)
Cu1—Cl3	2.2439 (11)
Cu1—Cl2	2.2495 (8)
Cu1—Cl4	2.2714 (8)

**Table:** 

Cl1—Cu1—Cl3	139.39 (3)
Cl1—Cu1—Cl2	99.91 (3)
Cl3—Cu1—Cl2	95.93 (3)
Cl1—Cu1—Cl4	96.75 (3)
Cl3—Cu1—Cl4	98.18 (3)
Cl2—Cu1—Cl4	134.61 (3)

**Table 2:** Hydrogen-bond geometry (Å, °)

*D*—H⋯*A*	*D*—H	H⋯*A*	*D*⋯*A*	*D*—H⋯*A*
N2—H2*C*⋯Cl4^i^	0.90	2.40	3.281 (2)	168
N1—H1*D*⋯Cl1^i^	0.90	2.43	3.282 (2)	157
N2—H2*B*⋯Cl3	0.90	2.37	3.2434 (19)	164
N1—H1*E*⋯Cl2	0.90	2.44	3.321 (2)	167

Symmetry code: (i) 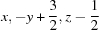.

**Fig. 1.**
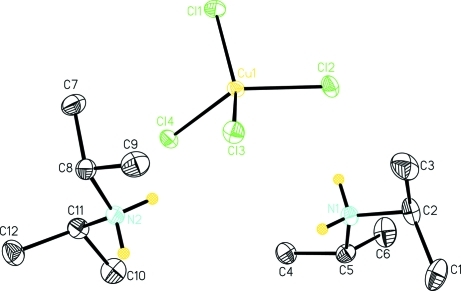
A view of the title compound with displacement ellipsoids drawn at the 30% probability level. C-bound H atoms have been omitted for clarity.

**Fig. 2.**
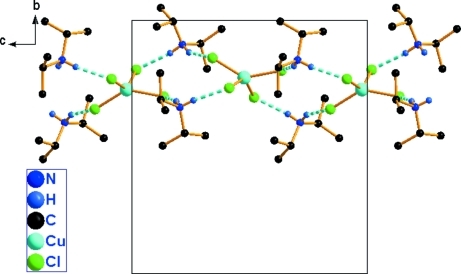
The crystal packing of the title compound viewed along the a axis showing the one-dimensionnal hydrogen bondings chain (dashed line). H atoms not involved in hydrogen bonding (dashed line) have been omitted for clarity.

## supplementary crystallographic information

### Comment

Simple organic salts containig amino cations have attracted an attention as materials which display ferroelectric-paraelectric phase transitions (e.g. Fu *et al.*, 2011). As part of our ongonig studies in this area, we now present the crystal structure of the title compound.

The asymmetric unit of the title compound contains two di-isopropylammonium cations and one CuCl_4_^2-^ anion (Table 1 and Fig. 1). Both the amine N atoms are protonated.

In the crystal structure, all the amino H atoms are involved in N—H···Cl H-bonding interactions with the Cl atoms of the CuCl_4_^2-^ anion with N···Cl distances between the range of 3.243 (2)Å to 3.321 (2)Å. These hydrogen bonds link the ionic units into a one-dimentional chain along the *c*-axis (Table 2 and Fig. 2).

### Experimental

A mixture of di-isopropylamine (0.8 mmol) and CuCl_2_ (0.4 mmol) were dissolved in HCl/EtOH/distilled water (1:1:1 *v*/*v*) solvent. The solution was slowly evaporated in air affording blue block-shaped crystals of the title compound.

### Refinement

The H atoms were geometrically placed (C—H = 0.96–0.98Å, N—H = 0.90Å) and refined as riding with *U*_iso_(H) = 1.2*U*_eq_(carrier) or 1.5*U*_eq_(C methyl).

### Crystal data

**Table:**

(C_6_H_16_N)_2_[CuCl_4_]	*F*(000) = 860
*M**_r_* = 409.74	*D*_x_ = 1.344 Mg m^−^^3^
Monoclinic, *P*2_1_/*c*	Mo *K*α radiation, λ = 0.71073 Å
Hall symbol: -P 2ybc	Cell parameters from 3479 reflections
*a* = 10.541 (2) Å	θ = 3.0–27.5°
*b* = 14.402 (3) Å	µ = 1.60 mm^−^^1^
*c* = 14.641 (6) Å	*T* = 298 K
β = 114.38 (2)°	Block, blue
*V* = 2024.5 (10) Å^3^	0.10 × 0.03 × 0.03 mm
*Z* = 4	

### Data collection

**Table:**

Rigaku Mercury2 CCD diffractometer	4637 independent reflections
Radiation source: fine-focus sealed tube	3904 reflections with *I* > 2σ(*I*)
Graphite monochromator	*R*_int_ = 0.036
Detector resolution: 13.6612 pixels mm^-1^	θ_max_ = 27.5°, θ_min_ = 3.1°
CCD profile fitting scans	*h* = −13→13
Absorption correction: multi-scan (*CrystalClear*; Rigaku, 2005)	*k* = −18→18
*T*_min_ = 0.910, *T*_max_ = 1.000	*l* = −18→19
20644 measured reflections	

### Refinement

**Table:**

Refinement on *F*^2^	Primary atom site location: structure-invariant direct methods
Least-squares matrix: full	Secondary atom site location: difference Fourier map
*R*[*F*^2^ > 2σ(*F*^2^)] = 0.035	Hydrogen site location: inferred from neighbouring sites
*wR*(*F*^2^) = 0.081	H-atom parameters constrained
*S* = 1.10	*w* = 1/[σ^2^(*F*_o_^2^) + (0.029*P*)^2^ + 0.869*P*] where *P* = (*F*_o_^2^ + 2*F*_c_^2^)/3
4637 reflections	(Δ/σ)_max_ = 0.001
180 parameters	Δρ_max_ = 0.50 e Å^−^^3^
0 restraints	Δρ_min_ = −0.41 e Å^−^^3^

### Special details

**Table:**

Geometry. All esds (except the esd in the dihedral angle between two l.s. planes) are estimated using the full covariance matrix. The cell esds are taken into account individually in the estimation of esds in distances, angles and torsion angles; correlations between esds in cell parameters are only used when they are defined by crystal symmetry. An approximate (isotropic) treatment of cell esds is used for estimating esds involving l.s. planes.
Refinement. Refinement of F^2^ against ALL reflections. The weighted R-factor wR and goodness of fit S are based on F^2^, conventional R-factors R are based on F, with F set to zero for negative F^2^. The threshold expression of F^2^ > 2sigma(F^2^) is used only for calculating R-factors(gt) etc. and is not relevant to the choice of reflections for refinement. R-factors based on F^2^ are statistically about twice as large as those based on F, and R- factors based on ALL data will be even larger.

### Fractional atomic coordinates and isotropic or equivalent isotropic displacement parameters (Å^2^)

**Table:**

	*x*	*y*	*z*	*U*_iso_*/*U*_eq_	
N2	0.48865 (18)	0.84283 (12)	0.29600 (13)	0.0392 (4)	
H2B	0.4207	0.8195	0.3114	0.047*	
H2C	0.4804	0.8158	0.2384	0.047*	
C8	0.4613 (2)	0.94547 (15)	0.27605 (18)	0.0452 (5)	
H8A	0.5328	0.9714	0.2569	0.054*	
C11	0.6264 (2)	0.81319 (18)	0.37713 (17)	0.0487 (5)	
H11A	0.6318	0.8358	0.4417	0.058*	
C9	0.3211 (3)	0.95517 (19)	0.1884 (2)	0.0620 (7)	
H9A	0.3002	1.0198	0.1737	0.093*	
H9B	0.2506	0.9273	0.2051	0.093*	
H9C	0.3233	0.9246	0.1308	0.093*	
C12	0.7457 (3)	0.8551 (2)	0.3585 (2)	0.0673 (8)	
H12A	0.7451	0.9213	0.3656	0.101*	
H12B	0.7350	0.8399	0.2919	0.101*	
H12C	0.8324	0.8306	0.4062	0.101*	
C10	0.6302 (3)	0.7088 (2)	0.3796 (2)	0.0698 (8)	
H10A	0.5538	0.6858	0.3927	0.105*	
H10B	0.7164	0.6883	0.4317	0.105*	
H10C	0.6229	0.6856	0.3162	0.105*	
C7	0.4685 (3)	0.99487 (19)	0.3691 (2)	0.0634 (7)	
H7A	0.5602	0.9880	0.4216	0.095*	
H7B	0.4014	0.9684	0.3901	0.095*	
H7C	0.4485	1.0596	0.3546	0.095*	
N1	0.09163 (18)	0.59594 (13)	0.28500 (13)	0.0411 (4)	
H1D	0.1413	0.6268	0.2574	0.049*	
H1E	0.0943	0.6299	0.3374	0.049*	
C2	−0.0577 (2)	0.5925 (2)	0.20865 (19)	0.0576 (7)	
H2A	−0.1146	0.5644	0.2400	0.069*	
C6	0.0892 (3)	0.4518 (2)	0.3773 (3)	0.0843 (10)	
H6A	−0.0008	0.4332	0.3287	0.126*	
H6B	0.0785	0.4906	0.4269	0.126*	
H6C	0.1423	0.3977	0.4091	0.126*	
C5	0.1649 (3)	0.50524 (16)	0.32533 (18)	0.0487 (6)	
H5A	0.1638	0.4680	0.2690	0.058*	
C4	0.3141 (3)	0.5256 (2)	0.3944 (2)	0.0600 (7)	
H4A	0.3572	0.5601	0.3588	0.090*	
H4B	0.3633	0.4683	0.4180	0.090*	
H4C	0.3169	0.5614	0.4505	0.090*	
C3	−0.1049 (3)	0.6917 (2)	0.1807 (2)	0.0803 (9)	
H3A	−0.0877	0.7268	0.2405	0.120*	
H3B	−0.2027	0.6926	0.1381	0.120*	
H3C	−0.0542	0.7187	0.1459	0.120*	
C1	−0.0719 (3)	0.5348 (2)	0.1193 (2)	0.0827 (10)	
H1A	−0.0459	0.4718	0.1401	0.124*	
H1B	−0.0120	0.5593	0.0904	0.124*	
H1C	−0.1667	0.5365	0.0704	0.124*	
Cu1	0.26640 (3)	0.773295 (18)	0.52212 (2)	0.03879 (9)	
Cl4	0.49483 (6)	0.73243 (4)	0.58638 (4)	0.05000 (15)	
Cl3	0.23772 (7)	0.80136 (4)	0.36402 (4)	0.05210 (15)	
Cl2	0.06532 (6)	0.69540 (5)	0.48106 (5)	0.05519 (16)	
Cl1	0.27415 (8)	0.85100 (5)	0.65701 (5)	0.06438 (19)	

### Atomic displacement parameters (Å^2^)

**Table:**

	*U*^11^	*U*^22^	*U*^33^	*U*^12^	*U*^13^	*U*^23^
N2	0.0416 (10)	0.0417 (10)	0.0380 (9)	−0.0027 (8)	0.0200 (8)	−0.0017 (8)
C8	0.0522 (13)	0.0385 (11)	0.0523 (13)	−0.0042 (10)	0.0289 (11)	−0.0003 (10)
C11	0.0464 (13)	0.0602 (15)	0.0355 (12)	0.0030 (11)	0.0128 (10)	0.0007 (11)
C9	0.0689 (18)	0.0498 (15)	0.0628 (16)	0.0123 (13)	0.0226 (14)	0.0078 (12)
C12	0.0449 (14)	0.082 (2)	0.0716 (19)	−0.0011 (14)	0.0208 (14)	−0.0004 (15)
C10	0.0741 (19)	0.0608 (17)	0.0659 (18)	0.0115 (14)	0.0201 (16)	0.0158 (14)
C7	0.0783 (19)	0.0520 (15)	0.0686 (18)	−0.0088 (13)	0.0390 (16)	−0.0174 (13)
N1	0.0389 (10)	0.0450 (10)	0.0401 (10)	−0.0018 (8)	0.0171 (8)	0.0045 (8)
C2	0.0355 (12)	0.0780 (19)	0.0547 (15)	−0.0065 (12)	0.0140 (11)	0.0168 (13)
C6	0.082 (2)	0.0666 (19)	0.096 (2)	−0.0093 (16)	0.0291 (19)	0.0345 (18)
C5	0.0543 (14)	0.0417 (12)	0.0470 (13)	0.0021 (10)	0.0179 (11)	0.0009 (10)
C4	0.0502 (15)	0.0638 (16)	0.0601 (16)	0.0099 (12)	0.0169 (13)	0.0094 (13)
C3	0.0601 (18)	0.096 (2)	0.071 (2)	0.0284 (17)	0.0131 (15)	0.0132 (18)
C1	0.073 (2)	0.085 (2)	0.0594 (18)	−0.0175 (17)	−0.0040 (15)	−0.0042 (16)
Cu1	0.04210 (16)	0.04234 (16)	0.03866 (15)	−0.00002 (11)	0.02342 (12)	−0.00011 (11)
Cl4	0.0416 (3)	0.0643 (4)	0.0472 (3)	0.0021 (3)	0.0214 (3)	−0.0004 (3)
Cl3	0.0613 (4)	0.0590 (3)	0.0436 (3)	−0.0059 (3)	0.0292 (3)	0.0079 (3)
Cl2	0.0426 (3)	0.0740 (4)	0.0546 (3)	−0.0075 (3)	0.0258 (3)	0.0014 (3)
Cl1	0.0727 (4)	0.0751 (4)	0.0618 (4)	−0.0067 (3)	0.0443 (4)	−0.0227 (3)

### Geometric parameters (Å, º)

**Table:**

N2—C11	1.509 (3)	N1—H1D	0.9000
N2—C8	1.511 (3)	N1—H1E	0.9000
N2—H2B	0.9000	C2—C1	1.504 (4)
N2—H2C	0.9000	C2—C3	1.513 (4)
C8—C7	1.510 (3)	C2—H2A	0.9800
C8—C9	1.511 (4)	C6—C5	1.519 (4)
C8—H8A	0.9800	C6—H6A	0.9600
C11—C10	1.505 (4)	C6—H6B	0.9600
C11—C12	1.517 (4)	C6—H6C	0.9600
C11—H11A	0.9800	C5—C4	1.507 (3)
C9—H9A	0.9600	C5—H5A	0.9800
C9—H9B	0.9600	C4—H4A	0.9600
C9—H9C	0.9600	C4—H4B	0.9600
C12—H12A	0.9600	C4—H4C	0.9600
C12—H12B	0.9600	C3—H3A	0.9600
C12—H12C	0.9600	C3—H3B	0.9600
C10—H10A	0.9600	C3—H3C	0.9600
C10—H10B	0.9600	C1—H1A	0.9600
C10—H10C	0.9600	C1—H1B	0.9600
C7—H7A	0.9600	C1—H1C	0.9600
C7—H7B	0.9600	Cu1—Cl1	2.2419 (9)
C7—H7C	0.9600	Cu1—Cl3	2.2439 (11)
N1—C5	1.508 (3)	Cu1—Cl2	2.2495 (8)
N1—C2	1.509 (3)	Cu1—Cl4	2.2714 (8)
			
C11—N2—C8	118.27 (18)	C5—N1—H1E	107.8
C11—N2—H2B	107.7	C2—N1—H1E	107.8
C8—N2—H2B	107.7	H1D—N1—H1E	107.1
C11—N2—H2C	107.7	C1—C2—N1	111.2 (2)
C8—N2—H2C	107.7	C1—C2—C3	112.5 (2)
H2B—N2—H2C	107.1	N1—C2—C3	107.1 (2)
C7—C8—C9	112.9 (2)	C1—C2—H2A	108.6
C7—C8—N2	110.6 (2)	N1—C2—H2A	108.6
C9—C8—N2	107.23 (19)	C3—C2—H2A	108.6
C7—C8—H8A	108.7	C5—C6—H6A	109.5
C9—C8—H8A	108.7	C5—C6—H6B	109.5
N2—C8—H8A	108.7	H6A—C6—H6B	109.5
C10—C11—N2	108.0 (2)	C5—C6—H6C	109.5
C10—C11—C12	112.7 (2)	H6A—C6—H6C	109.5
N2—C11—C12	110.2 (2)	H6B—C6—H6C	109.5
C10—C11—H11A	108.6	C4—C5—N1	108.54 (19)
N2—C11—H11A	108.6	C4—C5—C6	112.4 (2)
C12—C11—H11A	108.6	N1—C5—C6	110.2 (2)
C8—C9—H9A	109.5	C4—C5—H5A	108.5
C8—C9—H9B	109.5	N1—C5—H5A	108.5
H9A—C9—H9B	109.5	C6—C5—H5A	108.5
C8—C9—H9C	109.5	C5—C4—H4A	109.5
H9A—C9—H9C	109.5	C5—C4—H4B	109.5
H9B—C9—H9C	109.5	H4A—C4—H4B	109.5
C11—C12—H12A	109.5	C5—C4—H4C	109.5
C11—C12—H12B	109.5	H4A—C4—H4C	109.5
H12A—C12—H12B	109.5	H4B—C4—H4C	109.5
C11—C12—H12C	109.5	C2—C3—H3A	109.5
H12A—C12—H12C	109.5	C2—C3—H3B	109.5
H12B—C12—H12C	109.5	H3A—C3—H3B	109.5
C11—C10—H10A	109.5	C2—C3—H3C	109.5
C11—C10—H10B	109.5	H3A—C3—H3C	109.5
H10A—C10—H10B	109.5	H3B—C3—H3C	109.5
C11—C10—H10C	109.5	C2—C1—H1A	109.5
H10A—C10—H10C	109.5	C2—C1—H1B	109.5
H10B—C10—H10C	109.5	H1A—C1—H1B	109.5
C8—C7—H7A	109.5	C2—C1—H1C	109.5
C8—C7—H7B	109.5	H1A—C1—H1C	109.5
H7A—C7—H7B	109.5	H1B—C1—H1C	109.5
C8—C7—H7C	109.5	Cl1—Cu1—Cl3	139.39 (3)
H7A—C7—H7C	109.5	Cl1—Cu1—Cl2	99.91 (3)
H7B—C7—H7C	109.5	Cl3—Cu1—Cl2	95.93 (3)
C5—N1—C2	118.04 (19)	Cl1—Cu1—Cl4	96.75 (3)
C5—N1—H1D	107.8	Cl3—Cu1—Cl4	98.18 (3)
C2—N1—H1D	107.8	Cl2—Cu1—Cl4	134.61 (3)
			
C11—N2—C8—C7	−59.6 (3)	C5—N1—C2—C1	−56.3 (3)
C11—N2—C8—C9	176.88 (19)	C5—N1—C2—C3	−179.6 (2)
C8—N2—C11—C10	−177.9 (2)	C2—N1—C5—C4	176.3 (2)
C8—N2—C11—C12	−54.4 (3)	C2—N1—C5—C6	−60.3 (3)

### Hydrogen-bond geometry (Å, º)

**Table:**

*D*—H···*A*	*D*—H	H···*A*	*D*···*A*	*D*—H···*A*
N2—H2*C*···Cl4^i^	0.90	2.40	3.281 (2)	168
N1—H1*D*···Cl1^i^	0.90	2.43	3.282 (2)	157
N2—H2*B*···Cl3	0.90	2.37	3.2434 (19)	164
N1—H1*E*···Cl2	0.90	2.44	3.321 (2)	167

Symmetry code: (i) *x*, −*y*+3/2, *z*−1/2.
